# Cost per Event Averted in Cancer Trials in the Adjuvant Setting From 2018 to 2022

**DOI:** 10.1001/jamanetworkopen.2022.16058

**Published:** 2022-06-10

**Authors:** Idine Mousavi, Timothée Olivier, Vinay Prasad

**Affiliations:** 1 School of Medicine, New York Medical College, Valhalla; 2 Department of Oncology, Geneva University Hospital, Geneva, Switzerland; 3 Department of Epidemiology and Biostatistics, University of California, San Francisco, San Francisco

## Abstract

**Importance:**

Adjuvant therapies are often approved based on improvements in disease-, progression-, or relapse-free survival (ie, an event). An important estimate in adjuvant therapies is the cost per event averted.

**Objective:**

To characterize the costs per event averted of anticancer drugs approved by the US Food and Drug Administration (FDA) between January 2018 and March 2022.

**Design, Setting, and Participants:**

In this cross-sectional study, all approvals were reviewed from the FDA website. Approvals selected for the analysis needed to be drawn from randomized trials involving anticancer drugs studied in the adjuvant treatment of solid tumors. Treatments of nonsolid tumors; nonrandomized, noncontrolled trials; and unpublished trials were excluded. Approvals between January 2018 and March 2022 were included, and 11 trials met inclusion criteria. The monthly costs of each agent were abstracted from the Micromedex RED BOOK database. All variables included in calculations were derived from the original trial publication. Information abstracted for each approval included the name of the drug approved, name of the trial, primary end point, dosing regimen in the trial, median duration of treatment, tumor type, monthly costs per drug, and the number needed to treat for the primary end point. Data were analyzed in March 2022.

**Main Outcomes and Measures:**

The cost per event averted for each agent studied in each clinical trial; the monthly cost of each drug, the cost per patient for each drug, the primary clinical end point studied in each trial; the study’s design and setting.

**Results:**

A total of 11 approvals were included in the study. From January 2018 to March 2022, all approvals were based on a surrogate end point as the primary end point, with no trials demonstrating, to date, an overall survival benefit. The median cost per event averted of drugs in the adjuvant setting was $1 610 000 (range, $820 000 to $2 640 000). The median cost of a complete adjuvant treatment was $158 000 per patient.

**Conclusions and Relevance:**

In this cross-sectional study, anticancer agents in the adjuvant setting were approved based on disease-, progression-, event-, or relapse-free survival. Costs per event averted were high. These findings suggest that these drugs may not achieve wide or global use unless their efficacy improves or their prices decrease.

## Key Points

**Question:**

What is the cost per event averted among anticancer agents approved in the adjuvant setting?

**Findings:**

In this cross-sectional study of adjuvant anticancer US Food and Drug Administration drug approvals from January 2018 to March 2022, the median cost per event averted was $1 610 000 and the median cost per patient for an adjuvant treatment was $158 000.

**Meaning:**

These findings suggest that anticancer drugs in the adjuvant setting have considerable costs per event averted, raising questions of sustainability for patients and society.

## Teaser

This cross-sectional study evaluates the cost per event averted in adjuvant cancer trials between January 2018 and March 2022.

## Introduction

Globally, the price of cancer drugs is on an upward trajectory, with forecasts of global oncology therapeutic sales reaching $250 billion by 2024.^1^ In the US, the Centers for Medicare and Medicaid Services estimates that spending on health care will increase from USD $3.6 trillion in 2018 to approximately $6 trillion in 2027, approaching 20% of the gross domestic product, with the costs of cancer drugs playing a substantial role.^2^ The financial burden of these increasing costs falls upon patients and societies.^3^ Recent analyses^4,5,6^ have examined the association between the increasing costs of these anticancer agents and the benefits they deliver to patients. These analyses^4,5,6^ call into question whether increased costs translate directly into improved survival or improved quality of life for patients.

In contrast with the metastatic setting, where all patients have disease, drugs given in the adjuvant setting benefit only a fraction of treated patients, as some are already cured, and some will relapse despite treatment. As more patients are potentially eligible for adjuvant treatment than patients eligible for metastatic treatment, the budgetary impact of adjuvant therapy may profoundly impact global markets.

For this reason, we sought to assess the cost per event averted of different agents approved by the US Food and Drug Administration (FDA). We specifically aimed to characterize the monthly costs, the total cost for 1 patient’s treatment, and costs required to avert 1 negative outcome (cost per event averted) defined by each trial’s primary clinical end point.

## Methods

### Study Design and Search Strategy

This was a retrospective, cross-sectional study that sought all cancer-related randomized clinical trials on which an FDA approval was based in the adjuvant setting between 2018 and 2022. We adhered to the Strengthening the Reporting of Observational Studies in Epidemiology (STROBE) reporting guideline. We searched the FDA’s website for all approvals of oncology drugs according to clinical trials designed to study the drug in the adjuvant setting (ie, drugs given after primary resection of tumor) in all solid tumor types between January 2018 and March 2022. Searches were performed on March 15, 2022. We excluded trials that were noncontrolled, nonrandomized, or had not completed and published final trial analysis. Because we used publicly available data, and this is not human participants research in accordance with 45 CFR §46.102(f), we did not submit this study to an institutional review board or require informed consent procedures.

### Data Abstraction and End Points Calculation

Information abstracted for each trial included date of publication or approval, tumor type, setting, design (open or double-blind), phase of the trial, the primary trial end point, and overall survival data if available. The monthly cost of each drug was calculated on the basis of the mean wholesale price from the Micromedex RED BOOK database of drug pricing information. RED BOOK is a web-based drug pricing tool developed by IBM that reports prices sourced directly from drug manufacturers.

The cost of drug per patient was defined as the cost to complete 1 adjuvant treatment per patient. We calculated this cost assuming that patients received the reported median duration of treatment in the corresponding trial, and by multiplying the monthly cost of the drug by the median duration of treatment. From each publication, we calculated the number needed to treat from the difference in the Kaplan-Meier survival analysis at the latest time point as highlighted by the authors in the intention-to-treat population based on the study’s primary end point.

The cost per event averted was defined as the total cost in USD required to avert a defined negative outcome. Stated differently, it is the cost required to observe 1 beneficial event as defined by the specific clinical trial end point in the experimental group, as compared with the control group. We multiplied the cost of drug per patient by the number needed to treat to calculate the cost per event averted relating to the study’s primary end point. For an example of this calculation, please see the eAppendix in the Supplement.

### Statistical Analysis

Descriptive statistics were performed and reported throughout. Prices were rounded to the nearest 10 000 for estimates of cost per event averted and rounded to the nearest dollar for estimates of monthly cost to avoid artificial precision. All analyses were performed in Excel version 16.6 (Microsoft). Data were analyzed in March 2022.

## Results

We identified 11 trials^7,8,9,10,11,12,13,14,15,16,17^ in our analysis of adjuvant drugs that led to drug approvals between January 2018 and March 2022. The Table lists the name of each clinical trial, its design and clinical setting, its primary end point, the monthly cost of the drug, the cost per event averted per trial, and whether the trial showed an overall survival benefit at time of data publication, if reported. Because the intervention in the KEYNOTE 522 trial^16^ was both in the neoadjuvant and the adjuvant setting, this trial was included; no other neoadjuvant trial was included. There were 3 drug approvals^7,10,15^ for non–small cell lung cancer, 3 approvals^11,16,17^ for breast cancer, 2 approvals^8,13^ for melanoma, 1 approval^14^ for esophageal/gastroesophageal junction cancer, 1 approval^9^ for urothelial carcinoma, and 1 drug approval^12^ for kidney cell carcinoma. The sample size of the trials ranged from 682 to 1836 participants with a median sample size of 1019. Ten of 11 studies^7,8,9,10,11,12,14,15,16,17^ used a single investigational agent, and 1 study^13^ used 2 investigational agents (dabrafenib in combination with trametinib). Nine trials^7,8,9,10,12,13,14,15,17^ used a control group of observation or best supportive care, and 2 trials^11,16^ used a control group of chemotherapy. Nine trials^7,8,9,10,12,13,14,16,17^ had a double-blind design whereas 2 trials^11,15^ used an open-label design. These are also shown in the Table. The Figure shows a graphical representation of the cost per event averted for each trial, classified in an increasing order from bottom to top, with color-coding of the mechanism of action of drugs.

**Table.** Drug Cost per Event Averted in 11 Adjuvant Trials (2018-2022) According to RED BOOK Mean Wholesale Price Data

Study	Drug	Design and setting	Primary trial end point	Monthly cost of drug, $	Cost per event averted, $^a^	Overall survival benefit at time of analysis	Control group
EORTC 1325-MG/KEYNOTE-054,^8^ 2021	Pembrolizumab	Phase 3, double-blind, randomized; resected stage III melanoma	Recurrence-free survival	12 322	820 000	NA	Observation
CheckMate 274,^9^ 2021	Nivolumab	Phase 3, double-blind, randomized; muscle-invasive urothelial carcinoma	Disease-free survival	16 270	900 000	NA	Observation
PACIFIC,^7^ 2017	Durvalumab	Phase 3, double-blind, randomized; stage III NSCLC	Progression-free survival	15 713	910 000	NA	Observation
ADAURA,^10^ 2020	Osimertinib	Phase 3, double-blind, randomized; stage IB to IIIA *EGFR* variation–positive NSCLC after surgical resection	Disease-free survival	18 250	960 000	No	Observation
KATHERINE,^11^ 2019	Trastuzumab	Phase 3, open-label, randomized; *ERBB2*-positive breast cancer with residual invasive disease	Invasive disease-free survival	11 962	1 470 000	No	Chemotherapy
KEYNOTE-564,^12^ 2021	Pembrolizumab	Phase 3, double-blind, randomized; kidney carcinoma after nephrectomy	Disease-free survival	12 322	1 610 000	No	Observation
COMBI-AD,^13^ 2017	Dabrafenib	Phase 3, double-blind, randomized; stage III melanoma with *BRAF* V600E/K variations	Relapse-free survival	29 048	1 690 000	No	Observation
CheckMate 577,^14^ 2021	Nivolumab	Phase 3, double-blind, randomized; resected esophageal or gastroesophageal junction cancer	Disease-free survival	16 270	1 800 000	NA	Observation
IMpower010,^15^ 2021	Atezolizumab	Phase 3, open-label, randomized; resected stage 1B-IIIA NSCLC	Disease-free survival	11 364	2 230 000	No	Observation
OlympiA,^17^ 2021	Olaparib	Randomized, double-blind, phase 3; *ERBB2*–negative early breast cancer with *BRCA1/BRCA2* germline variations	Invasive disease-free survival	17 300	2 430 000	No	Observation
KEYNOTE-522,^16^ 2020	Pembrolizumab	Phase 3, double-blind, randomized; *HER2*–negative early breast cancer	Event-free survival	12 322	2 640 000	NA	Chemotherapy

Abbreviations: NA, not applicable; NSCLC, non–small cell lung cancer.

^a^ Median cost per event averted: $1 610 000.

**Figure.**
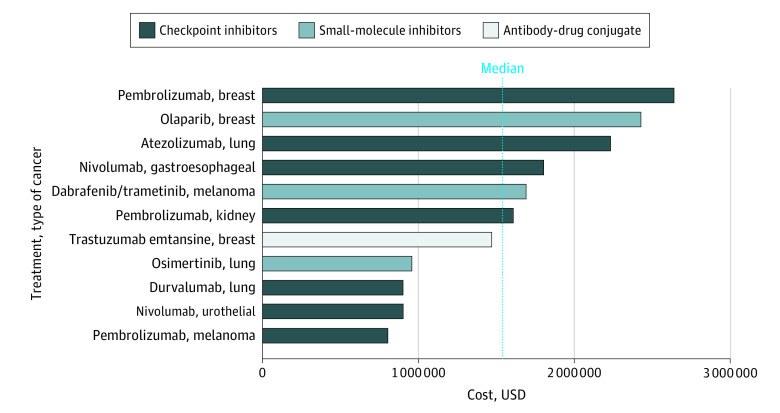
Cost per Event Averted of 11 Adjuvant Trials According to RED BOOK Mean Wholesale Price Data

The median (IQR) monthly drug cost was $16 000 ($12 322.46-$16 804.55) with a mean (SD) monthly cost of $15 700 ($5049.61). The maximum monthly drug cost of $29 048 was represented by dabrafenib and trametinib in the COMBI-AD trial study^13^ of stage IIIB melanoma with *BRAF* variants. The minimum monthly drug cost of $11 364 was represented by atezolizumab in the IMpower010 trial study^15^ of stage IB to IIIA non–small cell lung cancer.

We found the median (IQR) cost per treated patient to be approximately $158 000 ($149 101.77-$180 831.32) with a mean (SD) of $197 000 ($95 716.07). The maximum cost per treated patient was $440 000, represented by osimertinib in the ADAURA trial,^10^ whereas the minimum cost per treated patient was $118 000, represented by atezolizumab in the IMpower010 trial.^15^

The median (IQR) cost per event averted was $1 610 000 ($937 487.31-$2 019 844.56). The mean (SD) cost per event averted was $1 590 000 ($648 961.99). The maximum cost per event averted was $2 640 000, represented by pembrolizumab in the KEYNOTE-522 trial^16^ of early triple-negative breast cancer, whereas the minimum cost per event averted was $820 000, found in pembrolizumab in the KEYNOTE-054 trial.^8^ Disease-free survival was the most common primary end point, constituting the end point of 7 out of 11 trials.^9,10,11,12,14,15,17^ Overall survival analyses were performed in 6 of 11 trials.^10,11,12,13,15,17^ Of those 6, none have demonstrated a significant overall survival benefit at the time of this writing; however, the remaining trials have not yet reported final overall survival analyses because of data immaturity.

## Discussion

Our cross-sectional analysis of all US FDA approved anticancer drugs from January 2018 through March 2022 identified 11 randomized trials in solid tumors in the adjuvant setting. We found the median monthly cost was $16 000; and the median cost to avert 1 event as defined by the primary end point of the trial was $1 610 000, ranging from $820 000 to $2 640 000. All approvals were based on composite, surrogate end points (disease-, progression-, or relapse-free survival), and no agent had yet demonstrated an overall survival benefit at the date of our analysis.

Our study confirms the substantial monthly costs of newly approved therapies. Given the high cost of cancer drugs in the US and global markets, our analysis adds to the growing body of empirical data that points to the unsustainable costs of cancer drugs. For many patients with cancer, the high costs of treatment have substantial impacts on their financial solvency. Spending on anticancer drugs has increased, harming both patients and societies globally.^3^ Patients with cancer are more likely to file for bankruptcy than the general population with research pointing to bankruptcy as a factor associated with risk for early mortality in this population.^18^ The impact of these financial outcomes are strongly felt in low and middle income countries, where many patients have reduced access to treatments despite lower absolute costs compared with high income countries.

Although not FDA-approved as of the time of our analysis, and therefore excluded, our preliminary search included the CREATE-X trial of capecitabine in *ERBB2* (formerly *HER2*)–negative breast cancer.^19^ Interestingly, capecitabine in the CREATE-X trial was excluded from our analysis because it was not FDA approved in the setting of breast cancer. Capecitabine would have been the only drug available as a generic formulation and would have been the one with the lowest cost per event averted at $120 000. The absence of a branded sponsor may explain the lack of regulatory pursuit of this agent; notably, it is included in National Comprehensive Cancer Network guidelines and enjoys clinical use.

Coupled with financial toxic effects are the adverse effects of oncological agents. In most of the included trials, the control group was observation. As such, the impact on a patient’s quality of life with the addition of a therapeutic agent can only be detrimental. In other words, by definition, all adjuvant therapies make a patient feel worse than observation. These patients cannot have symptoms from cancer—as no cancer is detectable radiographically—and are taking medications to enhance their long-term prognosis.

The potential for life-threatening or long-lasting toxic effects from some agents, such as checkpoint inhibitors, must be noted in the context of the adjuvant setting.^20^ Concerningly, some patients never destined to relapse, or those that will inevitably relapse regardless of treatment, may experience adverse events from these drugs. Furthermore, time toxic events, meaning the global time spent to receive a treatment—such as drive time and infusion time—also places substantial demands on patients and their families.^21^

A drug can benefit a patient only if it is given to a patient. The lofty price to avert an event in our analysis hints that, even with steep discounts, many nations around the globe may have limited access to these agents in the adjuvant setting. No nation can consistently sustain treating patients with cancer with resected disease with agents that cost more than a million dollars per event averted.

All of the trials we examined defined the primary event as a composite time-to-event end point, such as disease-free survival, and none used overall survival. Although it is natural and intuitive to believe that gains in disease-free survival will inexorably translate into gains in overall survival, this assumption is uncertain. It is particularly uncertain for novel classes of medications that are already offered in advanced disease settings. For instance, some fraction of patients with non–small cell lung cancer may enjoy durable remission after programed cell death 1 antibody therapy. Is this fraction substantively higher if given in the adjuvant setting, or can the same fraction be rescued if the drug is given later, at metastatic relapse? Biology rather than volume of disease may be the defining event of programed cell death 1 sensitivity. Alternatively, a tyrosine kinase inhibitor may merely delay the time until inevitable recurrence. When recurrence occurs, the tumor may have exhausted sensitivity to the agent—as is potentially the case with anti–epidermal growth factor receptor therapy. Ultimately, whether adjuvant osimertinib is superior to osimertinib at relapse when given consistently remains an open and unresolved question. It is likely that the cost per event averted would be higher if an end point such as overall survival was defined as the primary outcome. Of the 10 trials included, 5 presented overall survival data at time of trial publication, with none having reached significance at the time of publication. While waiting for mature overall survival data, which will have to be interpreted regarding access to optimal postprogression therapy, our findings are in line with others that report a disconnection between the cost and efficacy of many oncology drugs.^4,5,6^

### Limitations

This study had limitations. First, the study period was limited. However, the study period encompasses more than 4 years and was designed to capture recent trends in adjuvant registration trials. Second, the calculation of number needed to treat was based on the availability of investigator-reported data at differing time points across trials, not allowing for direct comparisons between studies. Third, costs in the control group were not considered in the calculation; however, most trials used either placebo or observation as the control group. Furthermore, only the costs of the drug were considered, and costs associated with drug infusion, administrative costs, and labor costs were not included; our calculation could have, therefore, possibly underestimated the real cost per event averted.

## Conclusions

We found that all anticancer agents approved by the US FDA between January 2018 and March 2022 in the adjuvant setting were based on disease-, progression-, event-, or relapse-free survival. These approvals came with considerable costs per patient per treatment (median = $158 000) and high costs to avert 1 event (median = $1 610 000). Regulatory agencies and payers should ideally ensure that approved drugs provide clinically meaningful benefits with proportional and sustainable costs. Our analysis suggests that our current trajectory is unsustainable.

## Notes

Supplement. **eAppendix.** Cost per Event Averted Example Calculation
