# Supplementary material for: Cost per Event Averted in Cancer Trials in the Adjuvant Setting From 2018 to 2022
Source: JAMA Netw Open. 2022 Jun 10;5(6):e2216058. doi: 10.1001/jamanetworkopen.2022.16058 (PMC9187950; doi:10.1001/jamanetworkopen.2022.16058)
Supplement: Supplement. — eAppendix. Cost per Event Averted Example Calculation [file jamanetwopen-e2216058-s001.pdf]

Supplemental Online Content

Mousavi I, Olivier T, Prasad V. Cost per event averted in cancer trials in the adjuvant setting from 2018 to 2022. *JAMA Netw Open*. 2022;5(6):e2216058. doi:10.1001/jamanetworkopen.2022.16058

eAppendix. Cost per Event Averted Example Calculation

This supplemental material has been provided by the authors to give readers additional information about their work.

© 2022 Mousavi I et al. *JAMA Network Open.*

eAppendix: Cost Per Event Averted Example Calculation

- Example Calculation for Cost per Event Averted:
  - IMpower10 Trial - 3 yr (36 month) DFS endpoint (Intention-to-Treat population)
    - NNT = 1/ARR
      - % of ITT population in experimental group = 57.9%
      - % of ITT population in control group = 52.6%
    - NNT = 18.9
      - $1/(ER_{treatment} – ER_{control}) = NNT$
    - Median treatment duration = 10.4 months
    - Monthly cost of atezolizumab = $11,363.82
    - Cost per patient = (11,363.82*10.4) = $118,183.728
  - Cost per event averted = NNT*cost per patient = 18.9*118,183.728 = $2,233,672.4592 (rounded = **$2,230,000**)

© 2022 Mousavi I et al. *JAMA Network Open.*
